# Editorial: Self-care in healthcare workers for sustainable healthcare systems

**DOI:** 10.3389/fmed.2023.1190049

**Published:** 2023-04-18

**Authors:** Carolina S. Romero, Dietrich Doll, Amanda M. Kleiman, Markus M. Luedi

**Affiliations:** ^1^Department of Anaesthesiology and Critical Care, Hospital General Universitario de Valencia, Valencia, Spain; ^2^Department of Procto-Surgery, St. Marienhospital Vechta, Vechta, Germany; ^3^Department of Anaesthesiology, University of Virginia, Charlottesville, VA, United States; ^4^Department of Anaesthesiology and Pain Medicine, Inselspital, Bern University Hospital, University of Bern, Bern, Switzerland

**Keywords:** leadership, culture, conflict, burnout, purpose, self-management

The Severe Acute Respiratory Syndrome (SARS) Coronavirus 2019 (COVID-19) pandemic uncovered weak points in health care systems globally. Whereas technical dimensions were the bottleneck at the beginning (1–4), the availability of resilient personnel turned out to be a key problem in most health care systems in the course of the pandemic (5, 6).

With tremendously increased technical opportunities for effective patient care over the past decades, personal self-development often fell by the wayside and more and more of us are burning out (Tsai and Tsou; Grigorescu et al.). While specific settings might differ amongst different health care systems, the mechanisms of burnout are universal (Qureshi et al.) Challenging external demands that lead to an experience of detachment from one's job and a perceived lack of accomplishment finally lead to burnout (Che Yusof et al.) Health care systems globally urgently need new approaches for professional education toward sustainable self-care and respective staff development in health professions [(7, 8); Vidal-Alves et al.].

As originally biological and social beings, we humans have created a system that we no longer necessarily understand given the rapid technological developments, political turmoil, and progressive environmental destruction (Molero Jurado et al.). In addition, the discrepancy between the core of professions, i.e., to take care of people, and the privilege to be able to take care of ourselves is becoming more and more apparent as more and more healthcare workers struggle to find meaningful professional purpose to become the ideal version of ourselves.

As we learn from Peter Drucker, we do not have to change ourselves (Romero et al.), but we have to work hard to improve the way we perform. Effective self-care includes robust self-awareness to see beyond the daily drama, the ability to look into one self beyond the daily business and to control our thoughts, especially when facing high emotions (9). This implies to routinely “recharge batteries” by ensuring we meet our basic biological needs such as sleeping, exercising, and eating healthy foods (Sexton et al.). In addition, we must create truly human connections with ourselves and with the people (and patients) we lead (10). Recently, ancient contemplative practices have become as valuable resources in modern healthcare systems. In fact, the American Heart Association (AHA) states, that “there is increasing evidence that psychological health may be causally linked to biological processes and behaviors that contribute to and cause cardiovascular disease” (11) and “cancer” (12).

In this Research Topic about “*Self-care in healthcare workers for sustainable healthcare systems*” we present highly relevant studies to enrich health professions education globally. We shed light on recent developments in innovative human resource management practices and professional education about self-care and other non-technical skills amongst health professions to prevent burnout and ensure sustainable health care systems globally.

## Author contributions

CR, DD, AK, and ML helped to write the article and approved the final version. All authors contributed to the article and approved the submitted version.
